# Investigation of 222‐nm ultraviolet C irradiation bactericidal effect on the surgical field in a rabbit model

**DOI:** 10.1111/php.70031

**Published:** 2025-09-02

**Authors:** Tomoaki Fukui, Yuya Yamamoto, Kyohei Takase, Keisuke Oe, Kenichi Sawauchi, Ryota Nishida, Takahiro Niikura, Rena Kaigome, Masahiro Sasaki, Toru Koi, Hiroyuki Ohashi, Ryosuke Kuroda

**Affiliations:** ^1^ Department of Orthopaedic Surgery Kobe University Graduate School of Medicine Kobe Japan; ^2^ Department of Orthopaedic Surgery Hyogo Prefectural Nishinomiya Hospital Nishinomiya Japan; ^3^ Ushio Inc. Chiyoda‐Ku Tokyo Japan

**Keywords:** 222, rabbit, safety, ultraviolet C, UV‐C

## Abstract

Ultraviolet C (UV‐C) not only has a bactericidal effect, but is also cytotoxic; however, UV‐C at a wavelength of 222 nm with a high absorption coefficient for proteins is considered safe. We have previously reported the safety of 222‐nm UV‐C irradiation in humans and rabbits. This study evaluated the bactericidal effect of 222‐nm UV‐C irradiation on exposed surgical fields. Sixteen‐week‐old female rabbits were used, and the exposed area on their backs was sprayed with a bacterial solution from swabs collected from their soles. Three groups were formed based on UV‐C irradiation: 500 mJ/cm^2^ of 222‐nm UV‐C, 200 mJ/cm^2^ of 254‐nm UV‐C, which is commonly used in germicidal lamps, and non‐UV‐C irradiation. The bacterial colonies were counted after irradiation. Both UV‐C groups showed a significant reduction in bacterial colonies compared to the nonirradiated group, with no significant difference between the two UV‐C groups. Microbiota analysis identified species that could cause surgical site infections. The results of the study suggest that 500 mJ/cm^2^ of 222‐nm UV‐C irradiation effectively reduces bacterial load, with a bactericidal effect comparable to 254‐nm UV‐C; hence, 222‐nm UV‐C irradiation is a promising and safe tool for minimizing the risk of surgical site infections.

## Graphical abstract

In a rabbit surgical field model seeded with polymicrobial flora, a single dose of 222‐nm UV‐C (500 mJ/cm^2^) markedly reduced bacterial colonies, achieving a bactericidal effect comparable to 254‐nm UV‐C (200 mJ/cm^2^), while no UV‐C irradiation showed heavy growth. Wound healing did not differ among groups. Microbiota profiling detected SSI‐relevant taxa and showed culture‐reduced detected diversity. Because far‐UVC penetrates only microns into tissue, it supports intraoperative safety. 222‐nm UV‐C could be a promising adjunct to minimize surgical site contamination.

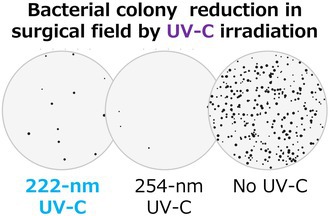

**Abbreviations**
CPD: cyclobutane pyrimidine dimer
DNA: deoxyribonucleic acid
MRSA: methicillin‐resistant Staphylococcus aureus
PBS: phosphate‐buffered saline
RNA: ribonucleic acid
SSI: surgical site infection
UV: ultraviolet

## INTRODUCTION

Advances in surgical treatments have brought great benefits to people; however, a certain percentage of complications occur during surgery, such as surgical site infections (SSI), which are serious complications. If an SSI becomes severe, it puts the life of the patient at risk; otherwise, it can lead to an extension of the treatment period and a decline in treatment outcomes.^1^ There are also concerns about the negative impact of SSI on medical economics.^2^ Therefore, resolving SSIs is extremely important for medical professionals, patients, and society. The pathogens responsible for SSIs include endogenous flora, namely indigenous skin bacteria and exogenous organisms such as airborne bacteria.^3^, ^4^ To prevent infection with these bacteria, a novel approach providing continuous intraoperative sterilization is warranted.

We previously performed a series of studies examining 222‐nm UV‐C as a new modality to prevent SSIs.^5^, ^6^ The primary rationale for focusing on 222‐nm UV‐C was its favorable safety profile, unlike that of 254‐nm UV‐C, used in conventional germicidal lamps,^7^ which has a high DNA absorption coefficient and is particularly toxic to human eyes and skin.^8^, ^9^, ^10^ The safety of 222‐nm UV‐C is attributed to its high protein absorption coefficient, which limits penetration through the stratum corneum.^11^, ^12^ Accumulated evidence supports the harmlessness of 222‐nm UV‐C; repeated, long‐term 222‐nm UV‐C irradiation did not induce skin cancer or ocular abnormalities in UV‐sensitive, tumor‐prone mice, whereas UV‐B exposure resulted in universal skin cancer, corneal damage, and keratitis.^13^ Using a methicillin‐resistant *Staphylococcus aureus* (MRSA)‐infected murine wound model, Narita et al. reported that 222‐nm UV‐C achieved efficacy comparable to or exceeding that of 254‐nm UV‐C.^14^

Our first study using 222‐nm UV‐C was a clinical experiment examining the safety and efficacy in healthy volunteers. We found that 500 mJ/cm^2^ of 222‐nm UV‐C irradiation was safe and bactericidal.^6^ As the next step in investigating the safety of 222‐nm UV‐C irradiation on tissues and organs not covered with skin, we conducted an experiment with 222‐nm UV‐C irradiation on several types of tissues that can be exposed in surgical fields, especially in orthopedic surgeries.^5^ To evaluate DNA damage by UV‐C, immunohistological assessment against cyclobutane pyrimidine dimer (CPD) was performed, and the CPD‐positive cell rate in 222‐nm UV‐C‐irradiated samples was significantly lower than that in 254‐nm UV‐C‐irradiated samples, indicating the safety of 222‐nm UV‐C irradiation on tissues without skin coverage. However, the bactericidal effect of 222‐nm UV‐C on the surgical field remains unknown.

Therefore, this study aimed to evaluate the bactericidal effect of 222‐nm UV‐C irradiation on exposed surgical sites in a rabbit model.

## MATERIALS AND METHODS

### Materials

Sixteen‐week‐old female New Zealand white rabbits (Japan SLC Inc., Hamamatsu, Japan) were used as experimental animals.

The experimental procedures adhered to the ethical principles set by the Animal Care and Use Committee of Kobe University Graduate School of Medicine, which granted approval for this study (approval no. P210204‐R1 and KM1010). The experiments were conducted in accordance with the Animal Research Reporting of In Vivo Experiments guidelines.

### Preparation of exposed area

The rabbits were anesthetized by inhalational anesthesia with 3–5% isoflurane (Fujifilm Wako Pure Chemical, Osaka, Japan) in 1.5–4.0 L/min of 100% O_2_ using a mask, followed by intraperitoneal injection of 0.5 mg/kg medetomidine (Nippon Zenyaku Kogyo, Koriyama, Japan), 2.0 mg/kg midazolam (Astellas Pharma, Tokyo, Japan), and 0.5 mg/kg butorphanol (Meiji Seika Pharma, Yokohama, Japan), and then a local injection of 30 mg/kg lidocaine hydrochloride (Aspen Japan, Tokyo, Japan) around the incision.

**FIGURE 1:**
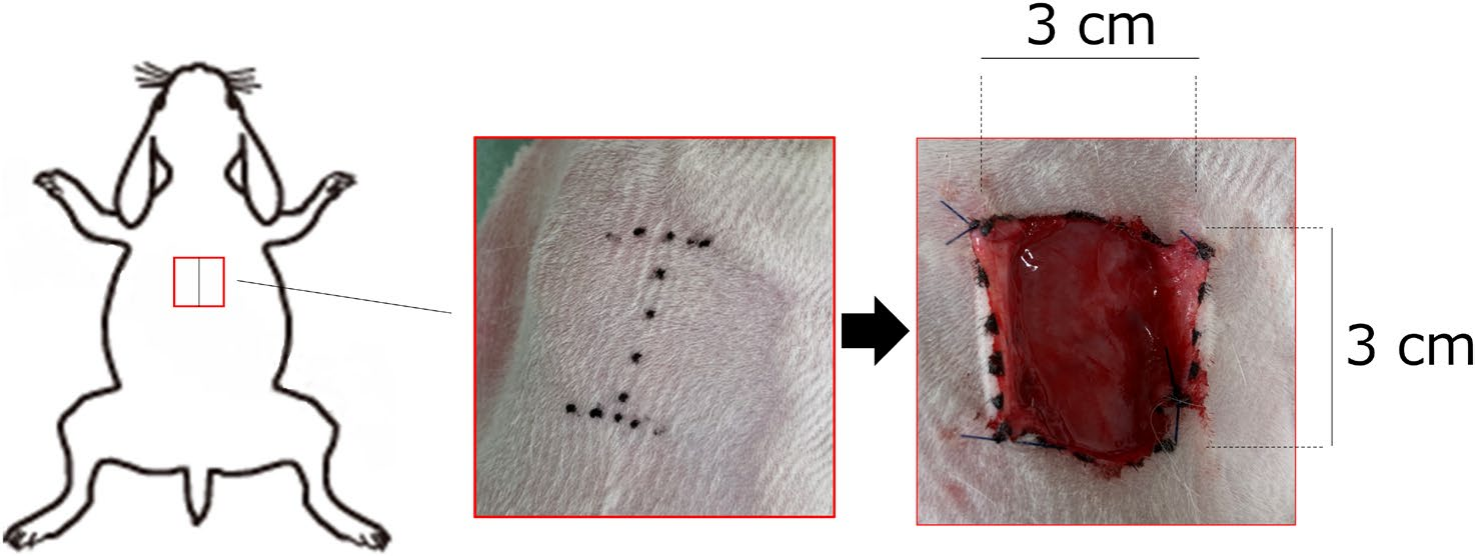
Macroscopic photographs of the irradiation area are shown. An H‐shaped incision, after shaving, was made and a subcutaneous tissue of 3 × 3 cm was exposed.

After shaving the dorsal hair, an H‐shaped skin incision was made to expose a 3 × 3 cm area of subcutaneous tissue, which was completely encompassed by the UV‐C irradiation field (Figure 1).

### Bacteria seeding on the exposed area

Both soles of each rabbit were scraped three times, using a dilution‐attached swab test (PBS; FK0101; Central Scientific Commerce, Inc., Tokyo, Japan), and swab solutions containing bacteria collected from the soles were sprayed three times onto the exposed area.

### UV‐C irradiation

Following our previous study,^5^ rabbits were randomly divided into three cohorts based on UV‐C exposure: 222‐nm UV‐C at 500 mJ/cm^2^ (222‐group); 254‐nm UV‐C at 200 mJ/cm^2^ serving as a positive control (254‐group); and no UV‐C irradiation as a negative control (control group; *n* = 10 per group). UV‐C irradiation was administered to the exposed tissue 5 min after bacterial seeding. For 222‐nm UV‐C irradiation, the SafeZoneUV‐C device (Ushio Inc. Tokyo, Japan), composed of a krypton‐chloride excimer lamp, air‐cooling fan, mirrors, and custom band‐pass filter blocking almost all wavelengths except the dominant 222‐nm emission wavelength,^6^ was used. Irradiance from the 222‐nm source was measured each time with an S‐172/UIT250 UV meter (Ushio Inc.), and exposure duration was calculated and adjusted based on those readings. The recorded irradiance was 4–5 mW/cm^2^. For 254‐nm UV‐C, a low‐pressure mercury lamp (FL‐4 W × 1; AS ONE, Osaka, Japan) was employed. Pre‐exposure irradiance measurements, performed as for the 222‐nm source, yielded 1 mW/cm^2^ in the irradiated region.

### Swab culture and colony number count

**FIGURE 2:**
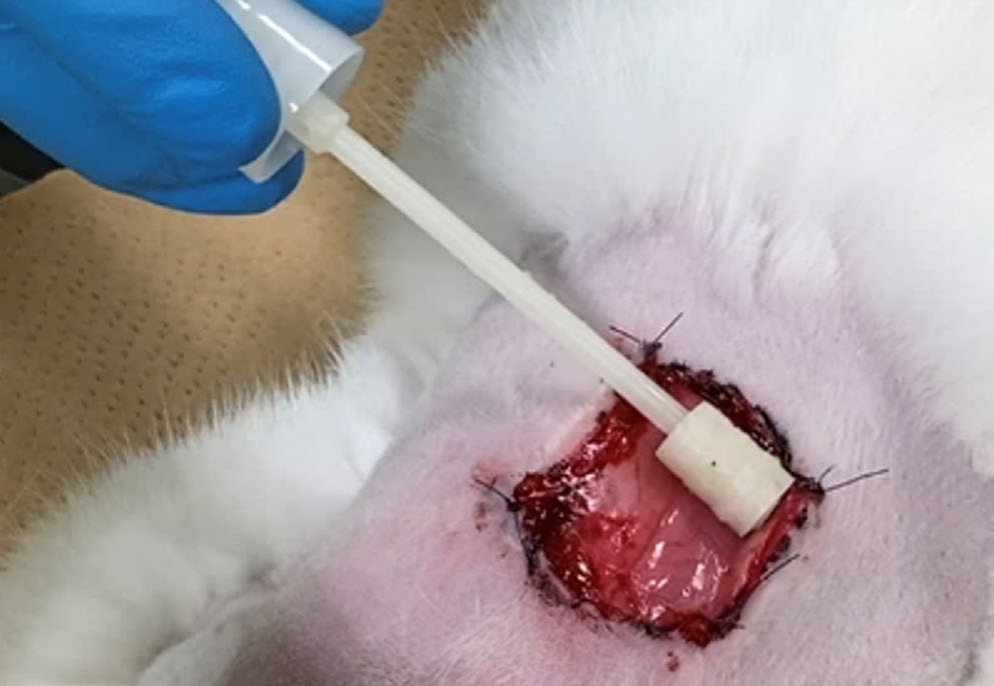
A macroscopic photograph of the exposed area after UV‐C irradiation or nonirradiation after scraping with a swab is shown. UV‐C, ultraviolet C.

The irradiated area was scraped with the same swab used to scrape rabbit soles (Figure 2). The swab diluent was filtered using a 0.45‐μm mixed cellulose ester membrane (EZ‐Fit filtration unit, Merck, Darmstadt, Germany). The membrane was then placed on soybean casein digest agar (Nissui Pharmacy, Tokyo, Japan) in a plastic dish and incubated at 37°C for 48 h. The colonies formed on the dishes were counted macroscopically. Membrane filtration was used because it was anticipated that swabbing a small exposed area would not yield a detectable number of bacteria.

### Skin closure and subsequent evaluation

The exposed area was closed using skin sutures after irradiation swabbing. One week after closure, the condition of the closed sites was evaluated macroscopically for wound healing, wound dehiscence, or infectious findings, such as pus discharge and swelling with redness.

### Microbiota analysis

In addition to the three groups, seven rabbits were subjected to microbiota analysis of the bacteria collected from the soles. The soles of all seven rabbits were wiped thrice using a dilution‐attached swab test (PBS). Seven swab solutions were mixed and diluted threefold with saline. The diluted swab solution was filtered and cultured at 37°C for 48 h, as described above. One third of the colonies from each of the three plates was collected and suspended in saline to create one sample. The samples were frozen at −20°C and subjected to microbiota analysis. This sample was used as the culture swab solution. To confirm the influence of the culture, the swab solution that was not cultured was also frozen and stored at −20°C, and subjected to microbiota analysis.

After thawing, the samples were mixed in the lysis buffer of the Maxwell® RSC Fecal Microbiome DNA Kit (Promega, WI, USA), and the entire volume of the buffer was transferred to an EZ‐Beads tube (Promega). Mechanical disruption was performed at maximum speed for 3 min using MM 400 (Retsch, Nordrhein‐Westfalen, Germany). After boiling for 5 min at 100°C, DNA purification was performed using magnetic beads with the Maxwell® RSC Fecal Microbiome DNA Kit. DNA was eluted with 50 μl of sterile water. DNA amplification targeting the V3‐V4 region of the 16S ribosomal RNA (rRNA) gene was performed by polymerase chain reaction using primers 341F and 806R. 16S rRNA gene sequencing was performed with reference to the 16S Metagenomics Sequencing Library Preparation (Illumina, USA).^15^ The obtained sequence reads were analyzed using QIIME 2 version 2023.2^16^ with the SILVA 138.1 SSU Ref NR 99^17^, ^18^ database.

### Animal euthanasia

The animals were euthanized using an overdose of sodium pentobarbital, following the procedures described above.

### Statistical analyses

The results were analyzed using GraphPad Prism (MDF Software, Inc.). The columns and error bars indicate the mean and standard error, respectively. The occurrence of wound dehiscence was analyzed using the chi‐squared test. Comparisons among the three groups were tested for significance using analysis of variance, followed by post hoc testing with Tukey's test. Statistical significance was set at *p* < 0.05.

## RESULTS

### Colony numbers detected after the irradiation

**FIGURE 3:**
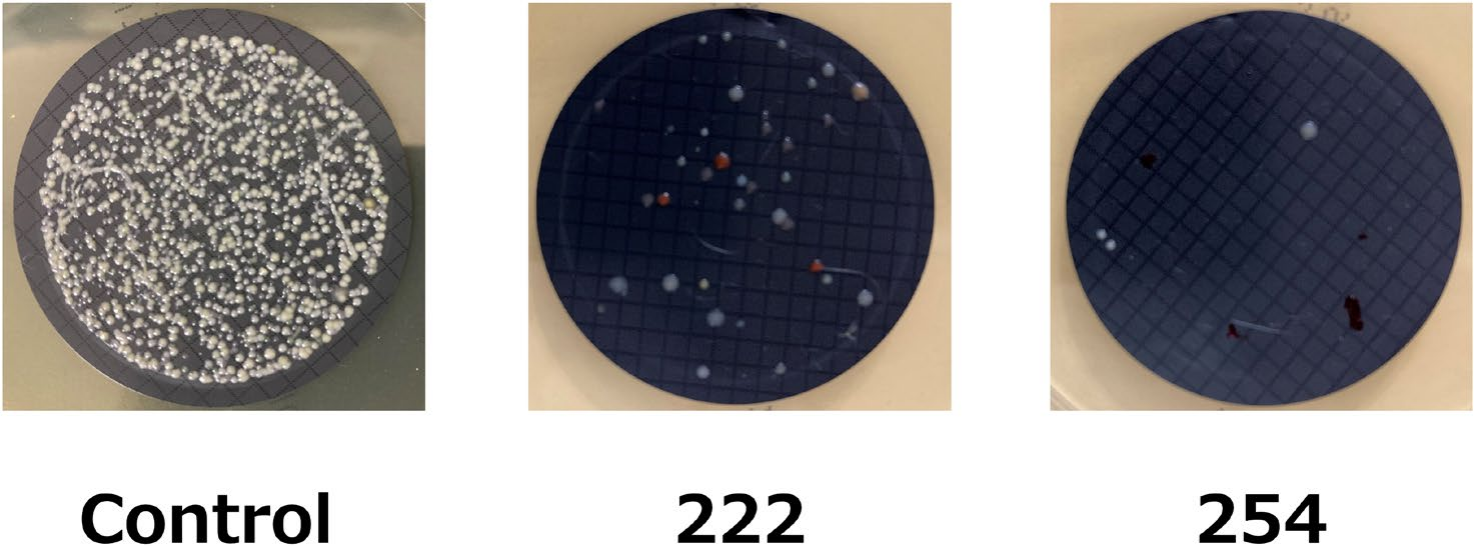
Representative macroscopic images of the membrane in the dishes of all three groups are shown. While the huge colonies are formed in the control group, the colonies in the 222‐ and 254‐groups look sparse.

**FIGURE 4:**
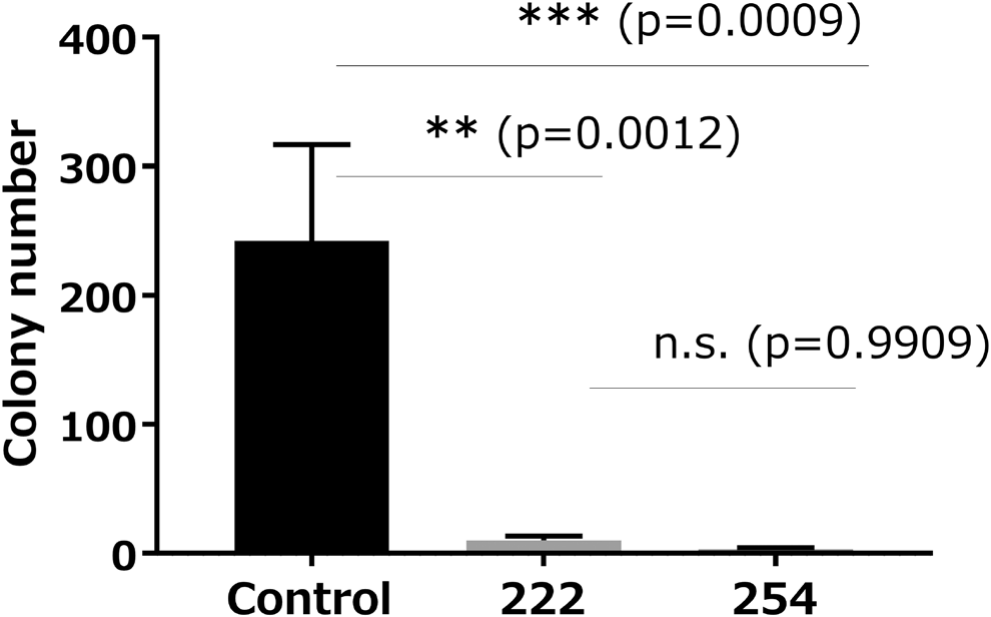
The mean number and standard deviation of the colonies formed in the groups are shown. Individual pairs of data are connected with lines. The colony number was significantly decreased in the 222‐ and 254‐groups compared to the control group.

The number of colonies formed in the control group was significantly higher than those in the 222‐ and 254‐groups (Figure 3). The colonies in one sample of the control group were too numerous to count; therefore, the sample was removed from the subjects and the remaining sample per group was as follows: control group, *n* = 9; 222‐group, *n* = 10; 254‐group, *n* = 10. The number of colonies in the control group was significantly greater than those in the 222‐ and 254‐groups. There was no significant difference between the 222‐ and 254‐groups (control, 242.0 ± 75.0; 222‐group, 10.2 ± 3.4; 254‐group, 3.0 ± 1.6; *p* = 0.0012 for control vs. 222‐group, *p* = 0.0009 for control vs. 254‐group, *p* = 0.9909 for 222‐group vs. 254‐group; Figure 4).

### Wound healing after irradiation

The wounds of all three groups (*n* = 10 each) were macroscopically evaluated 1 week after suturing. Wound dehiscence was observed in two, three, and three rats in the control, 222‐, and 254‐groups, respectively. The wounds of the other rats healed. None of the rats, including those with wound dehiscence, showed signs of infection, such as pus discharge or redness. There were no statistically significant differences in wound healing among the three groups (Table 1).

**TABLE 1:** The numbers of animals whose wounds are dehiscene or healed. There were no statistically significant differences in wound healing among the groups.

	Dehiscence	Healed
Control	2	8
222	3	7
254	3	7

### Analysis of microbiota

**FIGURE 5:**
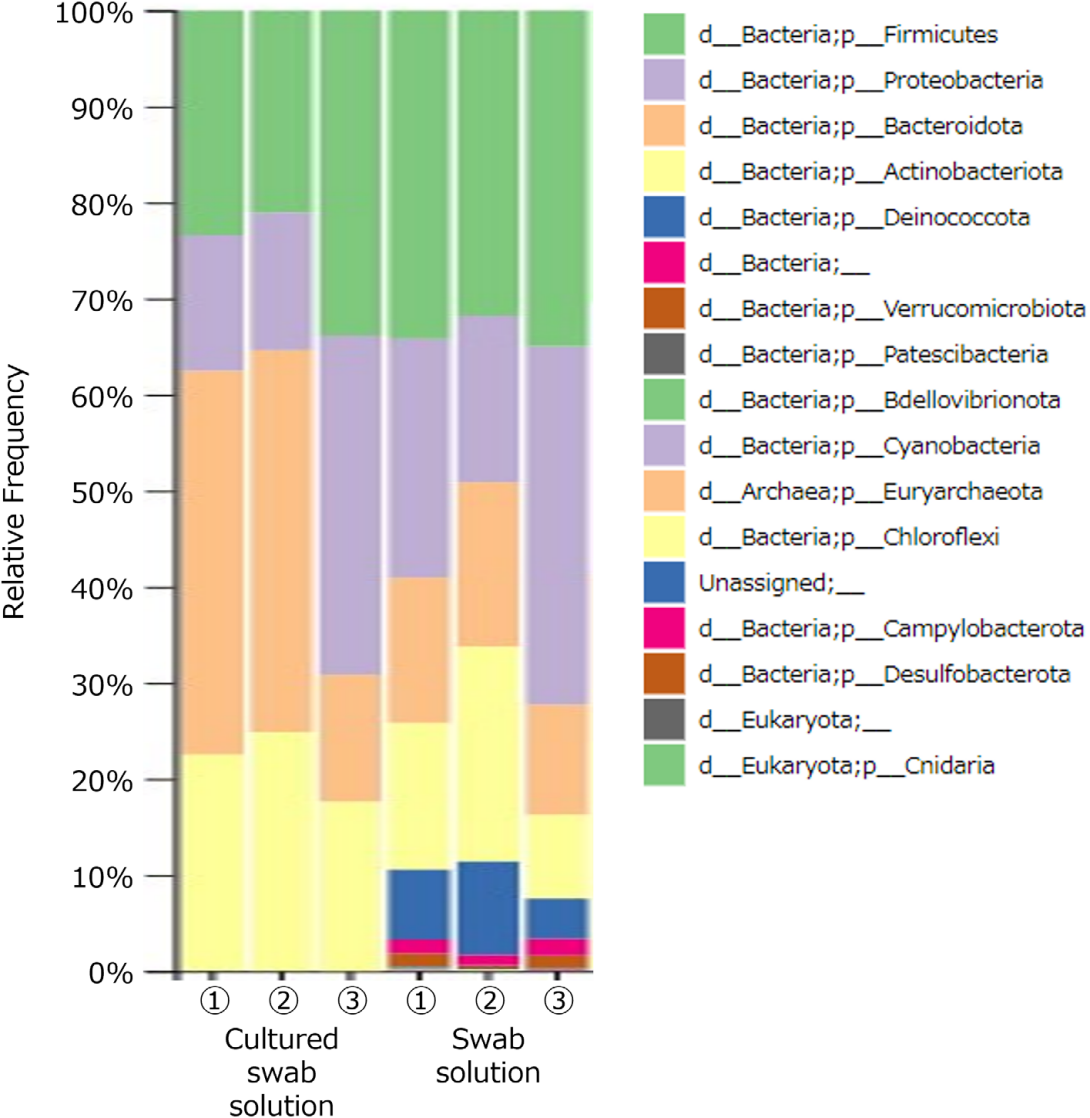
Microbiota analysis with phyla detected in the swab solutions and cultured swab solutions is shown. The legend indicates which phylum each color represents.

Database analysis showed that the microbiota in the foot sole was mainly composed of four phyla: Firmicutes, Bacteroidetes, Patescibacteria, and Actinobacteria. The following were identified in the cultured swab solutions: Firmicutes in 21–34%, Patescibacteria in 14–35%, Bacteroidetes in 13–40%, and Actinobacteria in 18–25%. In swab samples, Firmicutes were identified in 32–35%, Patescibacteria in 17–37%, Bacteroidetes in 12–17%, and Actinobacteria in 9–22%. Deinococcota were detected in approximately 10% of the swab solutions, but not in the cultured samples (Figure 5).

**FIGURE 6:**
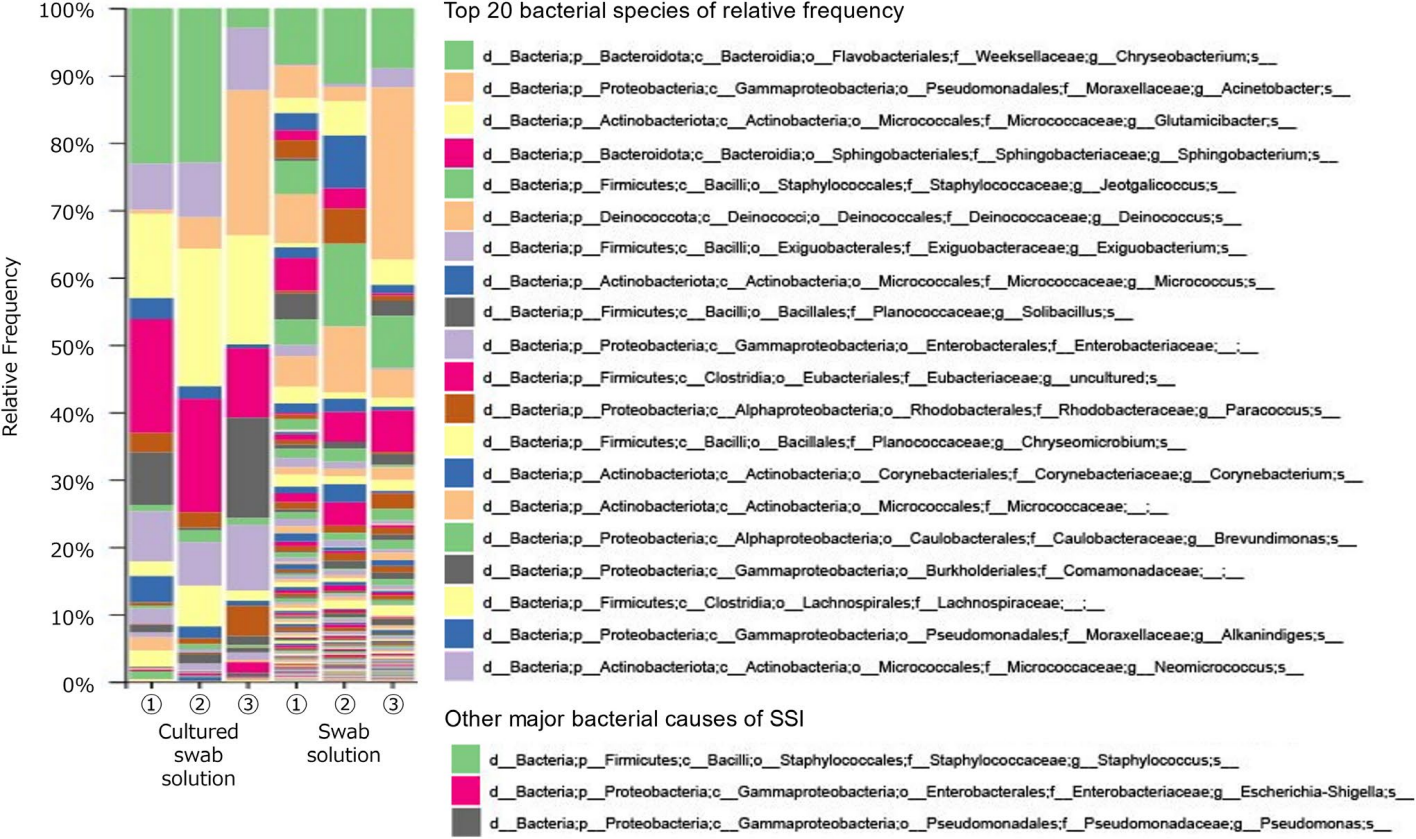
Microbiota analysis with the species detected in the swab solutions and cultured swab solutions. The legend indicates which species each color represents.

In an analysis using the SILVA database, four times more bacterial species were detected in non‐cultured swab solutions than in cultured swab solutions (Figure 6).

## DISCUSSION

This study was designed to rigorously evaluate the bactericidal effect of 222‐nm UV‐C irradiation on exposed surgical sites in a rabbit model to explore the potential of 222‐nm UV‐C as an innovative method for preventing SSIs, which remain a significant complication in modern surgical practice, including orthopedic surgeries. Our results indicated a substantial reduction in bacterial colony counts in tissues exposed to 222‐nm UV‐C, with an effectiveness that closely mirrors that of the traditionally used 254‐nm UV‐C. This outcome strongly suggests that 222‐nm UV‐C could serve as a potent novel method for reducing bacterial contamination during surgery, thereby lowering the risk of SSIs.

The idea of utilizing UV‐C to treat infection in surgeries has been around for a long time, and studies conducted at multiple hospitals have successfully used overhead UV‐C (254 nm) disinfection systems in operating rooms.^19^ This is a distinct advantage of UV‐C as a treatment for infection because it is less likely to cause resistance to antibiotics.^20^ In addition, antiseptics that are generally used on the skin preoperatively are cytotoxic when used in open wounds.^21^ Hence, UV‐C is superior as it does not cause any immediate local complications and has been proven to be cytotoxic in vitro.^22^

A previous study applied 254‐nm UV‐C irradiation to the field of orthopedic surgery, where UV‐C irradiation by an irradiator set on the ceiling of an operating room decreased the postoperative infection rate in almost 6000 total joint replacement surgeries.^23^ However, in the case of classic UV‐C light, such as the 254‐nm UV‐C used in germicidal lamps, concerns about its harmful effects on the human body remain. The 222‐nm UV‐C has recently attracted attention as a novel UV‐C that is safe for humans and is effective against various bacteria as well as viruses, including the cause of the coronavirus disease 2019, namely the severe acute respiratory syndrome coronavirus‐2.^23^, ^24^, ^25^, ^26^ The difference of safety to the human body between a UV‐C with a shorter wavelength (e.g., 222 nm) versus a longer wavelength occurs due to the difference in the absorbance coefficient of tissues, which depends on the wavelength.^11^ Shorter wavelength UV‐C, such as 222‐nm UV‐C, is more easily absorbed by proteins in the cytoplasm and is reduced to 50% by the tissue at 0.3 μm, in contrast to longer wavelength UV‐C, such as 254‐nm UV‐C, which is reduced to 50% by the tissue at 3 μm.^27^, ^28^ In addition, 222‐nm UV‐C only reaches the stratum corneum when radiated on human skin.^12^

Our group has previously established the use of 222‐nm UV‐C as a method for eliminating SSIs. The safety and bactericidal effects on the skin of healthy humans were investigated in a clinical trial. No erythema was induced by 222‐nm UV‐C irradiation at 50–500 mJ/cm^2^, whereas the minimum erythema dose of 254‐nm UV‐C has been reported to be 10 mJ/cm^2^. In addition, the number of bacteria collected from the skin was significantly decreased by 500 mJ/cm^2^ of 222‐nm UV‐C irradiation.^6^ These results indicate the safety and bactericidal efficacy of 222‐nm UV‐C on healthy skin and provide a rationale for the dosage of 222‐nm UV‐C applied in the current study. To examine the safety of tissues that are not covered with skin in the surgical field, in vivo studies were performed in rabbits.^5^ Five types of tissues that could appear in the surgical area of general orthopedic surgeries were surgically exposed and irradiated with 222‐ or 254‐nm UV‐C, and postirradiation changes were evaluated. DNA damage and apoptosis induced by 500 mJ/cm^2^ of 222‐nm UV‐C were similar to those induced by nonirradiation and were significantly lower than those irradiated by 254‐nm UV‐C, suggesting sufficient safety of 222‐nm UV‐C. In addition, the safety of 222‐nm UV‐C irradiation has been demonstrated in several other studies. Kaidzu et al. reported that no damage developed in the corneas of eyes exposed to 600 mJ/cm^2^ of 222‐nm UV‐C although damage, including keratitis and corneal epithelial erosion, was observed after 254‐nm UV‐C irradiation.^29^ A study using highly photocarcinogenic phenotype mice, which are highly sensitive to UV radiation and are approximately 10,000 times more likely to develop skin cancer than wild‐type mice, showed long‐term safety in the skin and eyes when exposed to 222‐nm UV‐C.^13^ Thus, evidence demonstrating the safety of 222‐nm UV‐C is accumulating.

The present study was conducted to verify whether 222‐nm UV‐C can exert a bactericidal effect in a surgical field not covered by the skin. To mimic airborne bacteria as a possible cause of SSI,^30^ we first attempted to induce infection in the surgically exposed field by leaving it open for 24 h. However, this did not work because only a small number of bacteria were observed (data not shown). Instead, bacteria were transferred from the soles where various bacteria existed. It was postulated that this model would primarily simulate SSIs caused by exogenous contamination such as airborne or contact‐transferred bacteria. The current results demonstrated an obvious and significant bactericidal effect of 500 mJ/cm^2^ of 222‐nm UV‐C, which is similar to that of 200 mJ/cm^2^ of 254‐nm UV‐C. An in vitro study reported that 222‐nm UV‐C can inactivate a wide range of pathogens, similar to 254‐nm UV‐C.^31^ However, since exudates usually exist in actual surgical sites and contain various types of proteins^32^ that can absorb UV‐C and decrease its bactericidal quality, unlike in vitro environments, it is necessary to perform in vivo studies to evaluate its efficacy as prophylaxis or treatment for SSIs. In an in vivo animal study, the bacterial count in mouse skin wounds following MRSA inoculation was significantly decreased by 222‐nm UV‐C irradiation compared to nonirradiation, and the bactericidal effect was comparable to that of 254‐nm UV‐C.^14^ While the previous study only examined MRSA, various kinds of bacteria were observed in the current study, which is a considerable difference between the two studies. Goh et al. conducted a clinical trial to determine the disinfection capabilities of 222‐nm UV‐C on human pressure ulcers and reported that it was effective in reducing bacterial colonies.^33^ Although the tissues exposed to pressure ulcers and the actual surgical field are different, our current results regarding efficacy are congruent with those of the previous study.

In the evaluation of the wound condition, there were no significant differences among the three groups, and no samples of infection were found. These findings suggest two conclusions. First, the current model is not an SSI model but a model to investigate the number of bacteria in an open field without skin. Second, UV‐C irradiation did not affect wound healing, and some wound dehiscence might have been due to problems with the suture procedure.

The analysis of microbiota in this study also provides valuable insights into the practical applications of 222‐nm UV‐C irradiation in surgical settings. The swab samples collected from the rabbit models revealed the presence of several bacterial phyla related to causative bacteria of SSIs in the orthopedic surgery field, namely *Enterococcus faecalis* classified in Firmicutes phylum, *Staphylococcus aureus* in Firmicutes, and *Pseudomonas aeruginosa* in Proteobacteria.^34^, ^35^ The fact that 222‐nm UV‐C was effective in significantly reducing the presence of these pathogenic bacteria in exposed tissues suggests that it could be an invaluable tool for the prevention of SSIs. However, it has been reported that the bactericidal effect of 222‐nm UV‐C may vary depending on the species^33^; nevertheless, the current microbiota analysis results did not detect specific species of pathogens commonly associated with SSIs such as *S. aureus* and *S. epidermidis*. This is a noteworthy concern and it would have been beneficial to determine the bactericidal effect of 222‐nm UV‐C on these species. Nonetheless, several previous in vitro studies already revealed its efficacy,^22^, ^36^, ^37^, ^38^, ^39^, ^40^ which could provide evidence for the clinical applicability of 222‐nm UV‐C in preventing SSIs. Moreover, considering that SSIs can involve complex polymicrobial communities, models based solely on single bacterial strains might lack external validity for evaluating bactericidal efficacy.^41^, ^42^ To this end, the use of polymicrobial flora in this study would enhance the clinical relevance of the model and better reflect real‐world contamination scenarios. Because the evaluation of colony number was performed via culture of the swab solution, microbiota analysis was performed for samples with or without culture. Interestingly, the types of detected species and phyla were decreased by the culture, although all samples contained bacteria that caused SSI; thus, it may be necessary to consider the influence of the culture when interpreting the results of cultured samples.

Despite these promising results, our study has some limitations that should be carefully considered when interpreting the findings. First, the study was conducted in a controlled laboratory environment using a rabbit model, which, while offering valuable insights, may not fully replicate the complex and variable conditions encountered in human surgical settings. Another important consideration is the variability in microbiota between different surgical sites and individual patients. While our study controlled for many variables, the diversity of bacterial species present in different clinical environments indicates that the effectiveness of 222‐nm UV‐C might vary in practice. In addition to these biological considerations, the practical aspects of the implementation of 222‐nm UV‐C in clinical practice must also be addressed. For instance, the design of the 222‐nm UV‐C devices for surgical use would need to be consistent and have adequate exposure to all relevant tissue surfaces. The potential for shadowing effects, where certain areas might not receive sufficient UV‐C exposure owing to obstruction by surgical instruments or anatomical structures, must be mitigated to ensure uniform sterilization.

In conclusion, this study presents compelling evidence that 222‐nm UV‐C irradiation could be a promising tool for reducing bacterial contamination at exposed surgical sites. However, to fully realize the potential of 222‐nm UV‐C in clinical practice, further research is needed to confirm its efficacy and safety in human subjects, as well as to explore its effects in a variety of clinical settings. By addressing these challenges and expanding our understanding of 222‐nm UV‐C irradiation, we can move closer to integrating this promising technology into standard surgical protocols, ultimately improving patient care and outcomes.

## Data Availability

All raw sequence data generated during this research are accessible from the DNA Data Bank of Japan under the accession number DRR727316‐DRR727321.
